# Prevalence of Relative Energy Deficiency in Sport (RED-S) among National Athletes in Malaysia

**DOI:** 10.3390/nu15071697

**Published:** 2023-03-30

**Authors:** Muhammad Irfan Haiqal Marzuki, Mohd Izham Mohamad, Wen Jin Chai, Nor M. F. Farah, Nik Shanita Safii, Jasmiza Khuzairi Jasme, Nor Aini Jamil

**Affiliations:** 1 Centre for Community Health Studies (ReaCH), Faculty of Health Sciences, Universiti Kebangsaan Malaysia, Kuala Lumpur 50300, Malaysia; 2 Sports Nutrition Centre, National Sports Institute of Malaysia, Bukit Jalil, Kuala Lumpur 57000, Malaysia; 3 Sports Medicine Centre, National Sports Institute of Malaysia, Bukit Jalil, Kuala Lumpur 57000, Malaysia

**Keywords:** relative energy deficiency in sport, low energy availability, sport, nutrition, athlete

## Abstract

Relative energy deficiency in sport (RED-S), which underpins the concept of low energy availability (LEA), can negatively affect athletes’ health and performance. This study aims to investigate the prevalence of RED-S among national athletes in Malaysia. A total of 192 national athletes (97 males, 18–39 years old) responded to an online survey regarding the RED-S risk. Most athletes (67.2%) were classified as having a medium/high RED-S risk. Female (41.2%), weight-class (23.9%), and intermittent sports (20.3%) had the highest prevalence of medium/high RED-S risk. Overall, most athletes spent 2–5 h daily (55.2%) and 6–7 days weekly (53.6%) practicing or playing their sport, and 47.9% reported feeling tired recently. The athletes were also terrified of being overweight (61.5%), worried about what they eat (64.7%), concerned about having fat on their bodies (62.6%), and thinking about burning calories while exercising (69.3%). Only 16.7% of the athletes had a prior history of stress fractures, and 40.6% continued to participate in activities 6–7 days per week while injured. The majority of female athletes (88.3%) had regular menstrual cycles. These findings support the notion that RED-S screening should be addressed in the athletic community. Future research is needed to clinically assess these athletes and investigate the impacts of RED-S on their health and sports performance.

## 1. Introduction

A good diet is necessary for athletes to meet their energy needs and attain their peak level of health and performance. Energy availability (EA) is the amount of energy available for normal metabolic function following exercise energy expenditure [1]. Low energy availability (LEA) develops when an individual or athlete consumes a low energy intake that is relative to expending a high exercise energy expenditure (EEE), which can occur intentionally in athletes who wish to lose weight or maintain a lower body weight through dietary restriction. LEA can also occur unintentionally in athletes due to an insufficient matching of one’s energy intake to meet energy needs during periods of high training volumes [2]. LEA is more common in athletes than in the general population due to the greater physical effort and physiological demands needed of athletes [3].

LEA can lead to relative energy deficiency in sport (RED-S), a condition that both male and female athletes may experience in an energy-deficient state [4]. LEA was first proposed in relation to the female athlete triad (FAT), as it is common in physically active females [4]. Disordered eating, amenorrhea, and osteoporosis are three distinct but interconnected components of FAT. The components were later termed energy availability, menstrual function, and bone mineral density, and each exists on a spectrum ranging from optimal health to disease [5]. However, as it is evident that relative energy deficiency affects many aspects of physiological function other than menstrual function and bone health, and the phenomenon also occurs in men, the International Olympic Committee (IOC) coined the term RED-S [2]. Recently, an official consensus statement on the male athlete triad was published, in which low testosterone or hypogonadism replaced menstrual dysfunction in the FAT [6].

RED-S can affect various health aspects, including the menstrual cycle, bone, hematologic, endocrine, and gastrointestinal health, which are all underpinned by LEA [7]. For sports performance, RED-S is associated with a poor training response, an increased injury risk, and decreased glycogen stores, muscle strength, and endurance performance [7]. Although it is unclear whether these implications manifest in a single system or across multiple systems, the consequences of RED-S are significant and detrimental to training, performance, and overall health [8,9].

Therefore, it is essential to identify athletes who may be at risk for RED-S for effective preventative and rehabilitation procedures to reduce the adverse effects of RED-S. Identifying and managing RED-S, however, remains difficult, despite a growing understanding of its causes and consequences. The most common methods to measure EA are food logs for energy intake and accelerometers or exercise logs for EEE, which are subjected to reliability and validity issues [10]. Validated questionnaires such as the Low Energy Availability in Females Questionnaire (LEAF-Q) and the RED-S clinical assessment tool (RED-S CAT) have been suggested for screening LEA and related physiological functions [11]. However, LEAF-Q is designed for female athletes only, whereas RED-S CAT requires specialized personnel, clinical facilities, and objective measurements of anthropometrics and biomarkers [12]. The Low Energy Availability in Male Athletes Questionnaire (LEAM-Q) was recently developed and validated in a large group of male athletes, but more research is required to expand the investigation of sex drive and other potential variables that are related to male athletes [13]. Regardless of the methods used, LEA and RED-S are still prevalent in male and female athletes [11,14,15,16].

Although Asian countries, including Malaysia, are seeing an increase in the number of people participating in sports, studies on RED-S are still lacking. Previously, a high prevalence of eating disorders (89.2%), menstrual irregularity (47.6%), and poor bone quality (13.3%) was reported among elite female Malaysian athletes in the lean sports group [17]. Currently, there has not been any published data on the prevalence of RED-S among Malaysian athletes. Therefore, this study aims to fill the research gap by identifying the prevalence of and those at risk for RED-S among national athletes in Malaysia. We hypothesized a high prevalence of RED-S risk in this sample of national athletes.

## 2. Materials and Methods

This cross-sectional study involved national athletes of various sports undergoing training at the National Sports Institute of Malaysia (NSI). We included athletes aged 18 and above who were Malaysian citizens and excluded paralympic and pregnant athletes. Ethical approval was obtained from the university’s Research Ethics Committee (reference code: UKM PPI/111/8/JEP-2022-303) and the NSI Research Ethics Committee (reference code: RE/A/008/2022-003/2022). All participants provided written informed consent prior to the study. Recruitment and data collection were conducted from April 2022 to September 2022.

The sample size was calculated using the Krejcie and Morgan (1970) equation for known population sample size [18]. Using the table value of Chi-square for 1 degree of freedom at the desired confidence level of 3.841(χ^2^), 0.05 degree of accuracy (d), with the population size of 428 athletes and assuming 50% of the population proportion (P), the calculated sample size was 205. Allowing for 10% of potential missing values and non-response rate, a total of 226 athletes were required.

An invitation with specific inclusion and exclusion criteria and a link to the online questionnaire (Google Form) was distributed to athletes via the WhatsApp application with the assistance of coaches and sports scientists at NSI. The questionnaire was bilingual (English and Malay). Data collected included sociodemographic profiles such as sex, date of birth, ethnicity, education level, height, weight, type of sport, project, and current training phase.

The RED-S-specific screening tool (RST), adapted and modified from Foley et al. (2020) [19], was used to assess the risk of RED-S. The modification included changes in the choices for special diet questions to ensure that they were culturally relevant. Specifically, we have reduced the options from 11 to 5 (no special diet, low carbohydrate, high protein, vegetarian, and others (please specify)). The questionnaire was back-translated into Malay, and a pilot study was conducted among 30 university athletes to assess its comprehension and readability. The findings showed a high level of understanding and acceptance with no difficulty. The internal consistency of the questionnaire was measured using Cronbach’s alpha. We performed the test for each sex and a combination of both sexes, excluding the menstrual function domain. Cronbach’s alpha was 0.76 for the male questionnaire, 0.80 for the female questionnaire, and 0.81 for both. Acceptable alpha values in the range of 0.70 to 0.95 have been proposed as good and reasonable [20].

The RST consists of 30 questions divided into seven domains: menstrual function (only for female participants), activity levels, nutrition and diet, injury, physiological effects, psychological effects, and factors affecting bone mineral density (BMD). Each component’s contribution to RED-S risk is weighted, yielding a total score of 880 for females and 730 for males. Athletes were classified as low-risk if their scores were less than 100 (males) or 150 (females), moderate-risk if between 101 and 400 (males) or 151 and 500 (females), and high-risk if greater than 400 (males) or 500 (females) [19].

The statistical data analysis was performed using IBM SPSS Statistics software version 26.0 (IBM SPSS Statistics Corporation, Chicago, IL, USA). Data were checked for normality using the Kolmogorov–Smirnov test, a histogram, and a scatterplot. Descriptive data were reported as the median and interquartile range (IQR) for continuous data and frequency and percentage for categorical data. Mann–Whitney U tests were used to compare the athletes’ physical characteristics, and the Chi-square test was used to compare their sociodemographic profiles. A Chi-square test of independence was used to determine any significant differences in potential RED-S risk between groups (sex, ethnicity, education level, sports category, training program, and training phase). The significance level was set at *p*-value < 0.05.

## 3. Results

**Figure 1:**
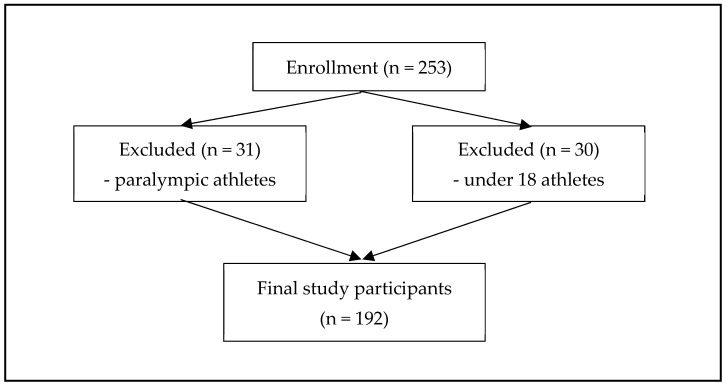
Recruitment of study participants.

A total of 253 athletes responded to the online survey (Figure 1). After excluding paralympic (*n* = 31) and under 18 (*n* = 30) athletes, the total number of participants included in this study was 192 (Table 1). The sports represented in the study were weight-class sports (27.6%), power sports (13.0%), intermittent sports (33.3%), endurance sports (4.7%), and skill sports (21.4%). More than half of the athletes (57.8%) were Malay, and the majority were part of the elite training program (74%). Overall, male athletes were taller and heavier than female athletes, and there were significant ethnic differences between the sexes. Other sociodemographic profiles showed no significant differences.

Table 2 presents the prevalence of RED-S among the participants. Overall, 67.2% of the athletes were classified as having a medium/high risk of RED-S. Specifically, about 65.1% were identified as having a medium risk, while 2.1% had a high risk of RED-S. Based on the sociodemographic profiles, female (41.2%), weight-class (23.9%), and intermittent sports (20.3%) had the highest prevalence of medium/high RED-S risk.

Further analysis revealed that most athletes spent 2–5 h daily (55.2%) and 6–7 days weekly (53.6%) training or playing their sports (Table 3). Nearly half of the athletes reported that they have not experienced any changes in their body weight recently (45.3%). However, more than half of the athletes reported that they were terrified of being overweight (61.5%), worried about what they eat (64.7%), concerned about having fat on their bodies (62.6%), and thinking about burning calories while exercising (69.3%). Other disordered eating behaviors, such as binge eating and following a special diet, were less common. Female athletes had more concerns about their body weight than male athletes, including being terrified of being overweight, being worried about having fat on their bodies, thinking about burning calories while exercising, feeling extremely guilty after eating, and wishing they were thinner (*p* < 0.05).

Only 16.7% of the athletes had a prior history of stress fractures, and nearly half (40.6%) continued participating in activities 6–7 days per week while injured (Table 4). When asked about their dairy consumption, 61% of the athletes reported that they drank milk either sometimes or never/rarely. Furthermore, 63% of the athletes reported not taking any calcium supplements. There were no significant differences in injury and factors affecting bone mineral density domains between the sexes. In terms of menstrual function, only 12.8% of female athletes reported primary amenorrhea. The vast majority (88.3%) had regular menstrual cycles.

Finally, nearly half of the athletes (47.9%) reported feeling tired recently, with female athletes reporting it more frequently, but the other physiological findings were unremarkable (Table 5). Over the previous six months, one-third of the athletes reported feeling stressed and nervous.

## 4. Discussion

To the best of our knowledge, this is the first study to present the prevalence of the relative energy deficiency in sport (RED-S) risk in Malaysian national athletes. Our findings highlight that more than half of the athletes (67.2%) were at risk of RED-S. These findings are consistent with previous studies among national to world-class middle/long-distance runners and race walkers (52.8%) [14], European cross-country athletes (64.3%) [15], and pre-professional, professional, and advanced amateur level dancers from 27 countries (54.2%) [16]. However, different studies used different methodologies to estimate the prevalence of LEA and RED-S since there is no gold standard of measurement [7].

Our present study showed that female athletes had a much higher risk of RED-S (41.2%) than male athletes (26%), which is consistent with other studies [11,14,15,16]. Specifically, female athletes were more concerned about their body weight than their male counterparts. Females were thought to be more vulnerable to LEA than males, as most studies on body dissatisfaction, restrictive dieting, and eating disorders focused on female athletes [21]. Furthermore, a more severe energy deficiency or LEA state is required to affect male reproductive and skeletal health compared to females [6,22]. However, further research is needed to clarify and quantify this relationship. There is growing evidence that LEA and RED-S are also prevalent in male athletes [23,24,25]. On the other hand, Beermann et al. (2020) found no gender differences in the risk of LEA in their study, which contradicts our findings [26]. This could be due to the fact that EA was assessed in their study using dietary diaries, whereas in our study, it was estimated using a questionnaire.

The findings also showed that athletes in weight-class (23.9%) and intermittent (20.3%) sports had the highest prevalence of RED-S risk compared to other sports categories. Several other studies found the same trend, with the prevalence ranging from 14% to 63% [27,28,29]. A specific level of leanness or weight is important in certain sports such as artistic gymnastics, diving, and weightlifting because it affects performance, appearance, and the need to compete in a specific weight category compared to athletes who participated in non-weight sports or where body weight was secondary [30]. In contrast to our findings, a study of collegiate national athletes and performing artists found no significant differences between LEA and sports categories [31]. Their study, however, was limited to female athletes.

The high prevalence of RED-S in this study can be attributed to the increased activity level. On average, more than half of the athletes trained 2–5 h per day, 6–7 days per week. Although almost half of them (47.9%) reported being more tired recently, it is unknown whether the athletes were exhausted due to their scheduled training or excessive exercise. Athletes participating in high-volume, intense training (e.g., 3–6 h per day, 5–6 days per week) may burn over 600–1200 calories per hour, which indicates a high exercise energy expenditure [32]. Since athletes who engage in excessive exercise are at risk of RED-S, future studies should investigate this association further.

When compared to the non-athlete population, athletes had a higher risk of experiencing reduced energy intake, which may result in an eating disorder (ED) or disordered eating (DE) [16,33,34,35]. In our study, 21.9% of the athletes reported losing weight recently, and the majority were terrified of being overweight, were thinking about burning calories while exercising, were worried about what they ate, and were concerned about having fat on their bodies. These factors, which are related to concerns about having higher levels of body fat, may lead to DE [36].

Our results showed that 29.2% of the athletes followed a special diet. All athletes are supported by sports scientists, including sports nutritionists and dietitians, at this institute. Each sports nutritionist/dietitian is assigned to a few sports, allowing for more personalized nutrition and close monitoring. Similarly, in a previous study (*n* = 1000) of high school and collegiate female athletes, about 23.4% of athletes reported following a specific diet [37]. In their study, athletes who followed a low-carbohydrate diet were more likely to report disordered eating than those without dietary restrictions. The fact that few athletes in the current study followed a special diet may also explain the low risk of anemia, although further biomarker analysis is required.

Only 16.7% of the athletes had a history of stress fractures, and nearly half (40.6%) continued participating in activities 6–7 days per week while injured. The number of athletes who reported having menstrual function issues, anemia, or psychological problems was low in this study. Cross-sectional studies of physically active female athletes with menstrual problems discovered that they were more likely to sustain bone stress injuries than athletes with normal menstruation [38]. Furthermore, performing activities frequently during an injury places excessive strain on the bones, therefore slowing the healing process. Most of the athletes also did not consume milk or calcium supplements regularly. A healthy diet that includes calcium and vitamin D is important for maintaining bone health [39,40].

This study adds to the body of knowledge about the prevalence of RED-S in national athletes participating in a variety of sports. One limitation of this study is the use of a self-report questionnaire, which relies on the athletes’ ability to comprehend and answer the questions truthfully. We modified the questionnaire, which required proper validation to determine its ability to screen for RED-S in our population. Future research should clinically assess the medium and high risk of RED-S athletes to determine its association with their sports performance and health.

## 5. Conclusions

A high prevalence of RED-S was identified among female, weight-class, and intermittent sports athletes. This may put athletes at risk for various physiological functions that could adversely affect their health and sports performance. With the acknowledged unfavorable health effects of RED-S, the study findings strengthen the case for recognizing this condition in the athletic community. In addition, it is crucial for athletes, coaches, and all sports personnel to be educated about the risk of RED-S and preventive measures. Future research is required to clinically assess these athletes and investigate the effects of RED-S on their health and sports performance, which will assist in RED-S management and treatment.

## Figures and Tables

**Table 1:** Physical characteristics and sociodemographic profiles of athletes.

	Male (*n* = 97)		Female (*n* = 95)		Total (*n* = 192)		
	Median (IQR)	*n* (%)	Median (IQR)	*n* (%)	Median (IQR)	*n* (%)	*p*-Value
Age (years)	22.7 (5.1)		22.3 (5.6)		22.7 (5.4)		0.431 ^a^
Weight (kg)	68.0 (12.0)		56.0 (11.0)		61.5 (15.0)		<0.001 ^a^
Height (cm)	170.0 (9.0)		160.0 (7.0)		165.0 (12.0)		<0.001 ^a^
**Ethnicity**							<0.001 ^b^
Malay		68 (70.1)		43 (45.3)		111 (57.8)	
Chinese		17 (17.5)		34 (35.8)		51 (26.6)	
Indian		4 (4.1)		5 (5.3)		9 (4.7)	
Others		8 (8.2)		13 (13.7)		21 (10.9)	
**Education level**							0.414 ^b^
Secondary school		49 (48.5)		37 (38.9)		86 (44.8)	
Pre-university		11 (11.3)		12 (12.6)		23 (12.0)	
Tertiary education		37 (38.2)		46 (48.4)		83 (43.2)	
**Sport category**							0.717 ^b^
Weight class		30 (30.9)		23 (24.2)		53 (27.6)	
Power		10 (10.3)		15 (15.8)		25 (13.0)	
Intermittent		33 (34.0)		31 (32.6)		64 (33.3)	
Endurance		4 (4.1)		5 (5.3)		9 (4.7)	
Skill		20 (20.6)		21 (22.1)		41 (21.4)	
**Athlete’s training program**							0.161 ^b^
Elite		76 (78.4)		66 (69.5)		142 (74.0)	
Development (junior)		21 (21.6)		29 (30.5)		50 (26.0)	
**Training phase**							
General preparation		27 (27.8)		25 (26.3)		52 (27.1)	0.183 ^b^
Specific preparation		24 (24.7)		16 (16.8)		40 (20.8)	
Pre-competition		27 (27.8)		27 (28.4)		54 (28.1)	
Competition		16 (16.5)		16 (16.8)		32 (16.7)	
Transition		3 (3.1)		11 (11.6)		14 (7.3)	

^a^ Mann–Whitney U tests; ^b^ Chi-square test.

**Table 2:** Prevalence of RED-S according to sociodemographic profile.

	Low RED-S Risk	Medium/High RED-S Risk	*p*-Value ^a^
	*n* (%)	*n* (%)	
**Sex**			<0.001
Male	47 (24.5)	50 (26.0)	
Female	16 (8.3)	79 (41.2)	
**Ethnicity**			0.051
Malay	45 (23.4)	66 (34.3)	
Chinese	10 (5.2)	41 (21.3)	
Indian	2 (1.0)	7 (3.6)	
Others	6 (3.1)	15 (7.8)	
**Education level**			0.561
Secondary school	30 (15.6)	56 (29.2)	
Pre-university	9 (4.7)	14 (7.3)	
Tertiary education	24 (12.5)	59 (30.7)	
**Sports category**			<0.001
Weight class	7 (3.6)	46 (23.9)	
Power	2 (1.0)	23 (11.9)	
Intermittent	25 (13.0)	39 (20.3)	
Endurance	3 (1.6)	6 (3.1)	
Skill	26 (13.5)	15 (7.8)	
**Athlete’s training program**			0.435
Elite	49 (25.5)	93 (48.5)	
Development (junior)	14 (7.3)	36 (18.7)	
**Training phase**			0.457
General preparation	19 (9.9)	33 (17.2)	
Specific preparation	14 (7.3)	26 (13.5)	
Pre-competition	19 (9.9)	35 (18.2)	
Competition	8 (4.2)	24 (12.5)	
Transition	3 (1.6)	11 (5.7)	

^a^ Chi-square test of independence.

**Table 3:** Responses on activity levels, nutrition, weight and diet domains.

Domain: Activity Levels	Responses	All		Sex		*p*-Value ^a^
		*n*	%	Male *n* (%)	Female *n* (%)	
How many hours in a day do you practice/play/do your sport/activity?	1–2 h	21	10.9	10 (10.3)	11 (11.5)	0.080
	2–5 h	106	55.2	61 (62.9)	45 (47.3)	
	5 h and above	65	33.9	26 (26.8)	39 (41.2)	
How many times in a week do you practice/play/do your sport/activity?	2–3 days a week	3	1.6	1 (1.0)	2 (2.1)	0.377
	3–5 days a week	86	44.8	48 (49.5)	38 (40)	
	6–7 days a week	103	53.6	48 (49.5)	55 (57.9)	
Do you feel like your ability to perform your sport has changed?	Yes	41	21.4	18 (18.6)	23 (24.2)	0.339
	No	151	78.6	79 (81.4)	72 (75.8)	
**Domain: Nutrition, weight, and diet**						
Have you recently had a change in your weight?	Yes, lost weight	42	21.9	24 (24.7)	18 (18.9)	0.620
	Yes, gain weight	63	32.8	31 (32.0)	32 (33.6)	
	No change	87	45.3	42 (43.3)	45 (47.5)	
Are you terrified about being overweight?	Usually/Always	49	25.6	15 (15.5)	34 (35.7)	0.007
	Sometimes	69	35.9	37 (38.1)	32 (33.6)	
	Never/Rarely	74	38.5	45 (46.4)	29 (30.7)	
Are you worried about what you eat?	Usually/Always	40	20.9	16 (16.5)	24 (25.2)	0.286
	Sometimes	84	43.8	42 (43.3)	42 (44.1)	
	Never/Rarely	68	35.3	39 (40.2)	29 (30.7)	
Are you worried about the thought of having fat on your body?	Usually/Always	63	32.9	32 (33.0)	31 (32.5)	0.044
	Sometimes	57	29.7	22 (22.7)	35 (36.8)	
	Never/Rarely	72	37.4	43 (44.3)	29 (30.7)	
Do you think about burning calories while exercising?	Usually/Always	75	39.1	38 (39.2)	37 (38.9)	0.048
	Sometimes	58	30.2	22 (22.7)	36 (37.8)	
	Never/Rarely	59	30.7	37 (38.1)	22 (23.3)	
Do you feel like you cannot stop eating, even if you feel full?	Usually/Always	14	7.3	6 (6.2)	8 (8.4)	0.776
	Sometimes	56	29.2	30 (30.9)	26 (26.3)	
	Never/Rarely	122	63.5	61 (62.9)	61 (65.3)	
Have you purposely thrown up after eating?	Sometimes	4	2.1	2 (2.1)	2 (2.1)	0.962
	Never/Rarely	188	97.9	95 (97.9)	93 (97.9)	
Do you feel extremely guilty after eating?	Usually/Always	11	5.8	4 (4.1)	7 (7.3)	<0.001
	Sometimes	40	20.8	15 (15.5)	25 (26.3)	
	Never/Rarely	141	73.4	78 (80.4)	63 (66.4)	
Do you wish you were thinner?	Usually/Always	45	23.5	15 (15.5)	30 (31.5)	0.004
	Sometimes	57	29.7	24 (24.7)	33 (34.7)	
	Never/Rarely	90	46.8	58 (59.8)	32 (33.8)	
Do you feel pressured by your friends, parents, or coaches to lose weight?	Usually/Always	9	4.7	4 (4.1)	5 (5.2)	0.134
	Sometimes	24	12.5	13 (13.4)	11 (11.5)	
	Never/Rarely	159	82.8	80 (82.5)	79 (83.3)	
Practice special diet	Low carbohydrate	19	9.9	6 (6.2)	13 (13.6)	0.405
	Vegetarian	9	4.7	6 (6.2)	3 (3.1)	
	High protein	28	14.6	13 (13.4)	15 (15.7)	
	No special diet	136	70.8	72 (74.2)	64 (67.6)	

^a^ Chi-square test of independence.

**Table 4:** Responses on injury and factors influencing BMD and menstrual function domains.

Domain: Injury and Factors That Affect BMD	Responses	All		Sex		*p*-Value ^a^
		*n*	%	Male *n* (%)	Female *n* (%)	
Have you ever had a stress fracture?	Yes	32	16.7	16 (16.5)	16 (16.8)	0.949
	No	160	83.3	81 (83.5)	79 (83.2)	
How much activity (hours/day) were you doing at the time of your injury?	0–1 h per day	9	28.1	5 (31.3)	4 (25.0)	0.335
	1–2 h per day	8	25.0	5 (31.3)	3 (18.7)	
	2–5 h per day	9	28.1	5 (31.3)	4 (25.0)	
	5 + hours per day	6	18.8	1 (6.1)	5 (31.3)	
How much activity (days/week) were you doing at the time of your injury (stress fracture)?	0–2 days per week	4	12.5	3 (18.7)	1 (6.1)	0.642
	2–3 days per week	5	15.6	3 (18.7)	2 (12.5)	
	3–5 days per week	10	31.3	4 (25.0)	6 (37.6)	
	6–7 days per week	13	40.6	6 (37.6)	7 (43.8)	
When you had the fracture, were you getting your period? (female athletes only)	No	10	31.3			
	Do not remember	6	18.8			
How often do you drink milk?	Usually/Always	75	39.0	41 (42.3)	34 (35.7)	0.100
	Sometimes	76	39.6	36 (37.1)	40 (42.1)	
	Never/Rarely	41	21.4	20 (20.6)	21 (22.2)	
Do you take calcium supplements?	Yes	71	37.0	41 (42.3)	30 (31.5)	0.125
	No	121	63.0	56 (57.7)	65 (68.5)	
**Domain: Menstrual function (female athletes only)**						
How old were you when you first got your period?	<15 years old	83	87.2			
	≥15 years old	12	12.8			
How often do you get your period?	More than once a month	5	5.3			
	Once a month	84	88.3			
	Once in 1–3 months	5	5.3			
	Less than every 3 months	1	1.1			
Are you prescribed any medication to help with your period or your hormones?	Yes	4	4.2			
	No	91	95.8			

^a^ Chi-square test of independence.

**Table 5:** Responses on physiological and psychological domains.

Domain: Physiological Effect	Responses	All		Sex		*p*-Value ^a^
		*n*	%	Male *n* (%)	Female *n* (%)	
Have you been told you have anemia?	Yes	14	7.3	5 (5.2)	9 (9.4)	0.511
	No	178	92.7	92 (94.8)	86 (90.6)	
Have you noticed a change in your skin color (i.e., have you become paler)?	Yes	9	4.7	6 (6.2)	3 (3.1))	0.321
	No	183	95.3	91 (93.8)	92 (96.9)	
Have you felt like you were going to faint?	Yes	33	17.2	11 (11.3)	22 (23.1)	0.030
	No	159	82.8	86 (88.7)	73 (76.9)	
Have you been more tired recently?	Yes	92	47.9	45 (53.6)	47 (49.5)	0.669
	No	100	52.1	52 (46.4)	48 (50.5)	
Fever/infection in the last 6 months	Yes	43	22.4	24 (24.7)	19 (20.0)	0.431
	No	149	77.6	73 (75.3)	76 (80.0)	
Do you have a heart condition?	Yes	2	1.0	1 (1.0)	1 (1.0)	0.988
	No	190	99.0	96 (99.0)	94 (99.0)	
**Domain: Psychological effect**						
Feelings that you have felt over the last 6 months	Easily annoyed	51	26.6	22 (22.6)	29 (30.5)	0.050
	Sad all the time	15	7.8	7 (7.2)	8 (8.4)	
	Hard to focus	53	27.6	29 (29.8)	24 (25.2)	
	Hard to make decision	51	26.6	27 (27.8)	24 (25.2)	
	Stressed	70	36.5	26 (26.8)	44 (46.3)	
	Nervous	65	33.9	31 (31.9)	34 (35.7)	

^a^ Chi-square test of independence.

## Data Availability

The data presented in this study are available on request from the corresponding author. The data are not publicly available due to ethical.
